# Intracardiac thrombosis after congenital heart disease surgeries in neonates: a report of two cases

**DOI:** 10.1186/s12887-023-04069-z

**Published:** 2023-06-02

**Authors:** Yanlin Yang, Jing Lv, Yajiao Li, Changping Gan, Peng Ji

**Affiliations:** 1 Department of Cardiovascular Surgery, West China Hospital, Sichuan University, No. 37 Guo Xue Alley, Wuhou District 610041, Chengdu, Sichuan Province China; 2 Anesthesia Operation Center, West China School of Nursing, West China Hospital, Sichuan University, Chengdu, Sichuan Province China; 3 Department of Cardiology, West China Hospital, Sichuan University, Chengdu, Sichuan Province China; 4 Department of Critical Care Medicine, West China Hospital, Sichuan University, No. 37 Guo Xue Alley, Wuhou District 610041, Chengdu, Sichuan Province China

**Keywords:** Congenital heart diseases, Critical care, Intracardiac thrombosis, Anticoagulation, Neonates

## Abstract

**Background:**

Intracardiac thrombosis (ICT) is a rare complication after the cardiopulmonary surgery for interrupted aortic arch (IAA) or total anomalous pulmonary venous connection (TAPVC) without previous records. There are still no general guidelines regarding as the mechanism or management of postoperative ICT in neonates and younger infants.

**Case presentation:**

We reported the conservative and surgical therapies in two neonates with intra-ventricular and intra-atrial thrombosis after the anatomical repair for IAA and TAPVC, respectively. There were no risk factors for ICT in both patients, except for the use of blood product and prothrombin complex concentrate. The surgery was indicated after TAPVC correction due to the worsening respiratory status and rapidly decreased mixed venous saturation. Anticoagulation combined with antiplatelet therapies was adopted in another patient. These two were both finally recovered, and three-month, six-month, and one-year follow-up echocardiography revealed no abnormality.

**Conclusions:**

ICT is uncommon in pediatric population after the surgery for congenital heart disease. Single ventricle palliation, heart transplantation, longer central line use, post-extracorporeal membrane oxygenation, and massive blood product use are major risk factors for postcardiotomy thrombosis. The causes of postoperative ICT are multifactorial, and the immaturity of thrombolytic and fibrinolytic system in neonates may serve as a prothrombotic factor. However, no consensus reached regarding as the therapies for postoperative ICT, and the large-scale prospective cohort study or randomized clinical trial is needed.

## Introduction

Intracardiac thrombosis (ICT) is not a common complication after the cardiopulmonary bypass (CPB) surgeries for congenital heart diseases. Cardiac anomaly per se and decreased function may promote a prothrombotic state, and even prothesis or implantable devices are also predisposed factors [1, 2]. However, ICT is rarely found in infants or neonates after the anatomical repair of total anomalous pulmonary venous connection (TAPVC) and interrupted aortic arch (IAA), which has mainly been documented in dilated cardiomyopathy, post-Norwood procedure, and single ventricle physiology [2–4]. We herein reported ICT in two neonates after CPB surgeries with different therapies, and the literature review was aimed to retrospect the potential causes, mechanisms, laboratory tests, and treatment strategies of ICT after congenital heart surgeries.

## Case reports

### Case 1

**Fig. 1:**
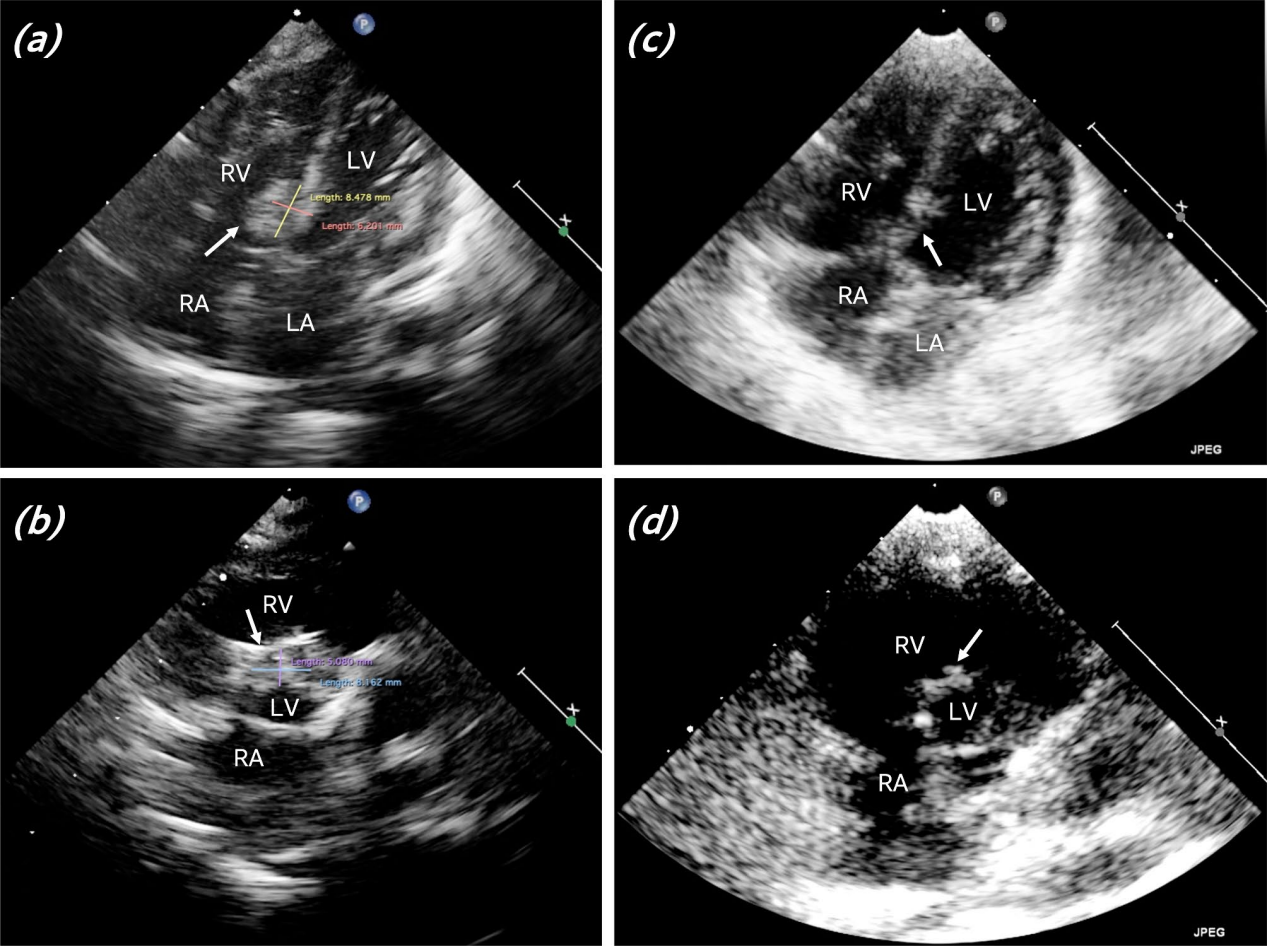
Apex five chamber (**a**) and parasternal short axis (**b**) views of TTE showing the ventricular septal defect patch (arrow) and the thrombus in the left ventricle with the maximum diameter of 8 × 6 mm. Post-treatment apex five chamber (**c**) and parasternal short axis (**d**) views of TTE showing the ventricular septal defect patch (arrow) without thrombus. LA, left atrium; RA, right atrium; LV, left ventricle; RV, right ventricle

A 24-day, 3.5-kilogram neonate was transferred to the emergency department for respiratory distress and feeding difficulty. The blood pressures of the right upper limb and the lower limb were 122/69 mmHg and 79/54 mmHg, respectively. Both lower extremities were pink, but no pulses were palpable. The transthoracic echocardiography (TTE) and computed tomography (CT) confirmed that the aortic arch was interrupted distal to the left subclavian artery and a large subaortic ventricular septal defect (VSD) coexisted. The patent ductus arteriosus (PDA) was nearly closed with the diameter of 2 mm. Alprostadil was initiated to maintain the PDA. Emergency surgery was performed under moderate hypothermic cardiac arrest (MHCA) with selective cerebral perfusion. The distal aorta was anastomosed to the proximal arch and the VSD was closed with a bovine pericardial patch. The operation was uneventful, and TTE on postoperative day (POD) 1 illustrated unobstructed aortic arch, well closed VSD, and good heart function. The hemostatic components, including prothrombin complex concentrate (PCC) and fibrinogen, and blood transfusion were routinely used after neonatal MHCA surgery according to our center’s practice. Unexpectedly, TTE on POD 4 detected a thrombus (6⋅8 mm, Fig. 1a-b) adhering to the left side of VSD patch without any hemodynamic compromise or signs of thrombosis. The enoxaparin sodium and aspirin were given at the dose of 170 AXaIU/kg (0.017 ml/kg) twice a day and 5 mg/kg per day, respectively. The peak anti-factor Xa concentration was monitored at the level of 0.5 ~ 1.0 IU/ml to ensure the efficacy of anticoagulation. The left ventricular (LV) thrombus resolved a week after the anti-coagulation and anti-platelet therapy (Fig. 1c-d) without any signs of embolism. No emerging ICT was observed during 6-month follow-up.

### Case 2

**Fig. 2:**
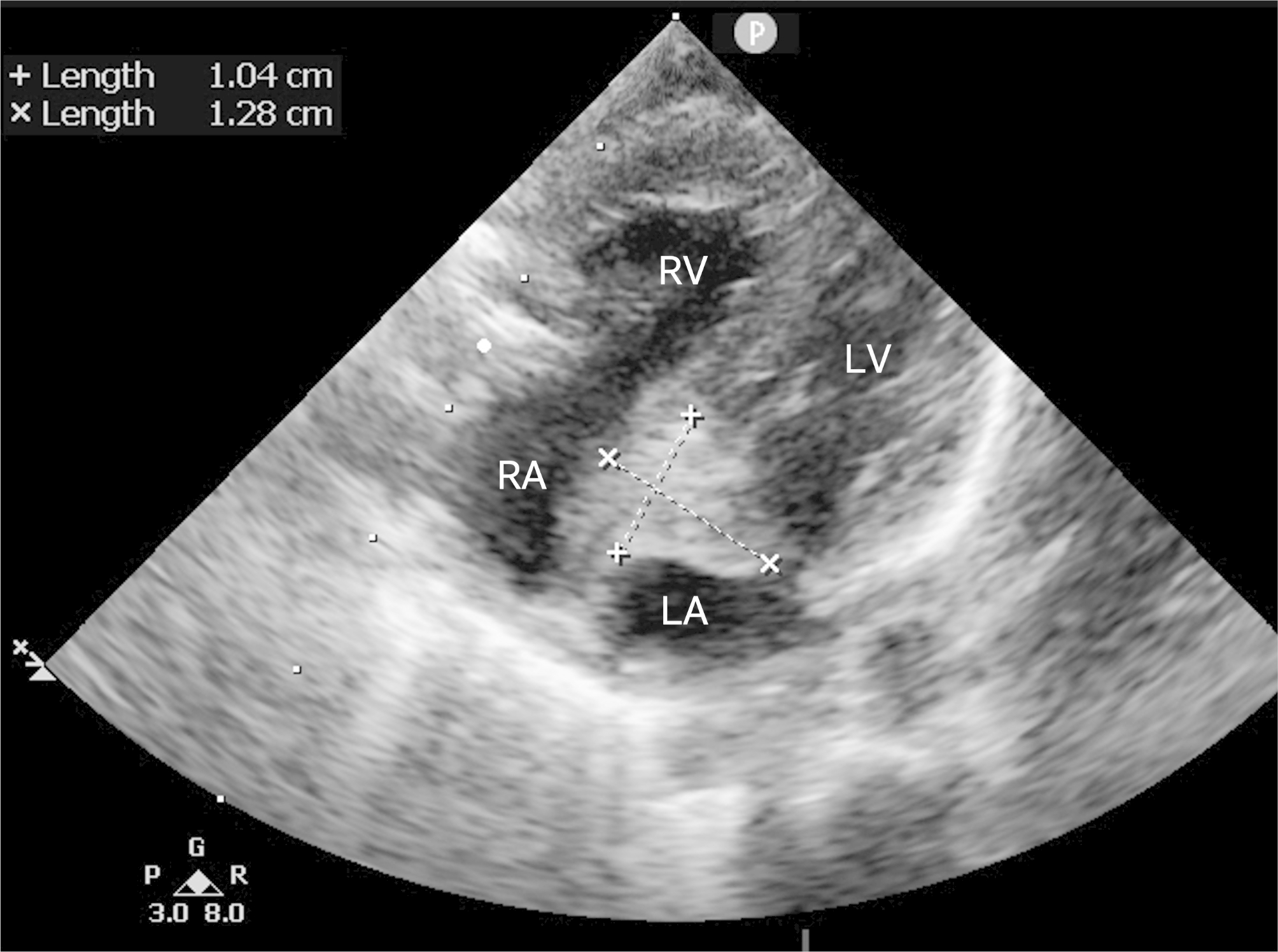
Apex four chamber view of TTE showing the intraatrial thrombus attached to the right side of the atrial septal defect patch. LA, left atrium; RA, right atrium ; LV, left ventricle; RV, right ventricle

A 2.8-kilogram newborn was admitted to our center due to uncorrectable cyanosis after delivery. TTE discovered four pulmonary veins converging into a common trunk which drained into the right atrium through dilated coronary sinus, and the small atrial septal defect (ASD) was restrictive. CT scan illustrated the diagnosis of intracardiac TAPVC. The anomaly was uneventfully repaired under CPB in a usual fashion, in which the veins were baffled into the left atrium by a bovine pericardial patch. With the protocol, neither anticoagulation nor antiplatelet therapy were used. However, the chest drainage increased in the first postoperative 24 h, and we used prothrombin complex concentrate (PCC) and fibrinogen for the hemostatic purpose. Respiratory distress was observed on POD 5, and mixed venous saturation dropped from 65 to 50% within 6 h. The patient was re-intubated and TTE surprisingly revealed a large area of thrombosis (10⋅12 mm, Fig. 2) on both sides of the patch. The level and function of the platelet were both normal. The screen of coagulation factors and antithrombin III (ATIII) found no congenital abnormalities. Owing to the deteriorated condition, an emergency surgery was performed. Massive thrombosis on both sides of the bovine pericardial patch was removed, and the invasiveness of the thrombosis to the pulmonary vein area explained the clinical picture. The original patch was abandoned, and the atrial septum was reconstructed with a new bovine pericardial patch. The hemodynamic status was stable after the second surgery and there was no abnormal occupying found on echo afterwards. No signs of perioperative brain injury or thromboembolism were recorded. Three-month and one-year follow-up did not show relapse of the ICT.

## Discussion and conclusion

ICT is rare in neonates after cardiac surgeries. A few cases of left ventricular thrombi were reported after the Norwood operation, repair of anomalous left coronary artery from the pulmonary artery, or aortic stenosis with severely reduced LV contractility [5–7]. However, no reports mentioned any similar situation after surgical repair of IAA or TAPVC, for which we usually believe that the risk of ICT is minimal and anticoagulation therapy is not needed. Although most pediatric intracardiac thrombus was treated with anticoagulation therapy and the thrombus would usually resolve, we still described a necessary and successful surgical intervention for an atrial thrombus with hemodynamic turbulence.

Postoperative ICT could occur at all kinds of location in clinical practice, corresponding to a variety of triggers and causes [1, 2]. However, the causes of thrombosis in both cases were unknown. In case 1, the thrombus was in LV, which may be contributed to the subaortic concave geometry change close to the VSD patch [8]. The shift of hemodynamics, left heart compliance, LV systolic function, and abnormal ventricular wall motion after the aortic surgery could not be ignored, either. K Hanseus et al. blamed the LV thrombus in a newborn with severe aortic stenosis on the severely abnormal hemodynamics [7]. W Duncan et al. presumed that the formation of LV thrombus was due to the ongoing flow stasis within LV in a hypoplastic left heart syndrome after Norwood surgery [5]. LV function may play an important role in the thrombosis, and even a short period of LV dysfunction may lead to thrombus formation. The obstructive thrombus was at the atrial level in case 2, in which the first operation focused on the atria. The use of central venous catheter (CVC) was also one of the risk factors, but no signs of thrombosis on CVC was detected. We speculated that a mass of collagen components of the endocardium exposed after incision of the atrial tissue could result in thrombosis. Therefore, the suturing closure of endocardial margin after the unroof of the coronary sinus is recommended in this type of procedure [9, 10]. Although the coronary sinus wasis always baffled to the left atrium, the thrombus formed in the right atrium in case 2, which indicated other risk factors participating in the formation of ICT.

Neonates are at risk of thrombosis after surgical trauma due to hypercoagulability, which might be caused by lower levels of anticoagulants and immaturity of antifibrinolytic system [4, 11, 12]. Injury of the vascular wall and blood stasis during the operation also contribute to the postoperative hypercoagulable status. It is well-known that increased factor VIII (FVIII), protein C (PC), protein S (PS), plasminogen activator inhibitor-1 (PAI-1), and thrombin-activatable fibrinolysis inhibitor (TAFI), and decreased ATIII are associated the high risk of thrombosis after cardiac surgery [13, 14]. Mutations of factor V Leiden and prothrombin (G20210A), as well as methylenetetrahydrofolate reductase (MTHFR) polymorphism, result in congenital abnormalities of coagulation and fibrinolytic system, and further influence thrombosis after the surgery [15, 16]. Regrettably, the thrombin generation assay (TGA) or immunoassays of coagulants and antifibrinolytic components were not regularly tested in both cases, while spontaneous thrombosis or hemorrhage were not recorded in the follow-up. This might indirectly imply the absence of congenital disorders.

The PCC and fibrinogen were used in both patients after surgeries (Table 1), which might serve as a prothrombotic factor. However, it was concluded that PCC was deemed to be safe and effective for postoperative bleeding in children and infants after cardiac surgeries, and no thrombosis complications were reported during ICU stay [17, 18]. In fact, current guidelines still suggest that the risk of thrombosis should be weighed against the risk of hemorrhage before the use of PCC [19, 20]. Another potential risk factor may be the patch material as both thrombi sticked to the septal patches. In fact, we used to apply the bovine pericardial patch to other neonatal cases with coarctation of the aorta plus VSD, in which the pathophysiology and postoperative condition are similar to IAA/VSD; however, we haven’t seen any comparable complications so far. It was also demonstrated that there were no differences on the manifestation between autologous and xeno-pericardium in published articles [21, 22].

**Table 1:** Comparison of the clinical features between patients suffering from intracardiac thrombosis with IAA and TAPVC.

	Case 1	Case 2
**Preoperative characteristics**		
Age at surgery (day)	26	17
Weight at surgery (kilogram)	3.5	2.8
Premature	N	Y
Primary diagnosis	IAA (Type-A)	intracardiac TAPVC
Combined anomaly	PDA, VSD	PDA, ASD
Congestive heart failure	N	Y
**Intraoperative characteristics**		
Surgical procedure	IAA repair, PDA ligation, VSD closure	TAPVC repair, PDA ligation, ASD closure
CPB strategy	Moderate hypothermia (24–28 ℃) cardiac arrest with selective cerebral perfusion	Mild hypothermia (32–34 ℃)
CPB duration (min)	155	67
Aortic clamp time (min)	87	31
Bovine pericardial patch use	Y	Y
**Postoperative characteristics**		
Arrhythmia	N	N
Delayed sternal closure	Y	N
Highest lactate (mmol/L)	1.5	6.7
Blood product use	RBCs 2U	RBCs 1U, leukocyte-reduced RBCs 1U, plasma 200ml
Hemostatic drug use	Prothrombin complex concentrate 150U, fibrinogen 50 mg/kg	Prothrombin complex concentrate 150U, fibrinogen 50 mg/kg
Anticoagulation treatment	Enoxaparin*	Enoxaparin*
Antiplatelet treatment	Aspirin*	N
First 24-hour drainage (ml)	200	80
First 48-hour drainage (ml)	267	150
PT on POD 1 (s)	20	22
APTT on POD 1 (s)	46	39

*IAA, interrupted aortic arch; TAPVC, total anomalous pulmonary venous connection; PDA, patent ductus arteriosus; VSD, ventricular septal defect; ASD, atrial septal defect; CPB, cardiopulmonary bypass; RBCs, red blood cells; PT, prothrombin time; APTT, activated partial thrombin time.*

*Enoxaparin and aspirin were used after the intracardiac thrombosis was detected.

**Fig. 3:**
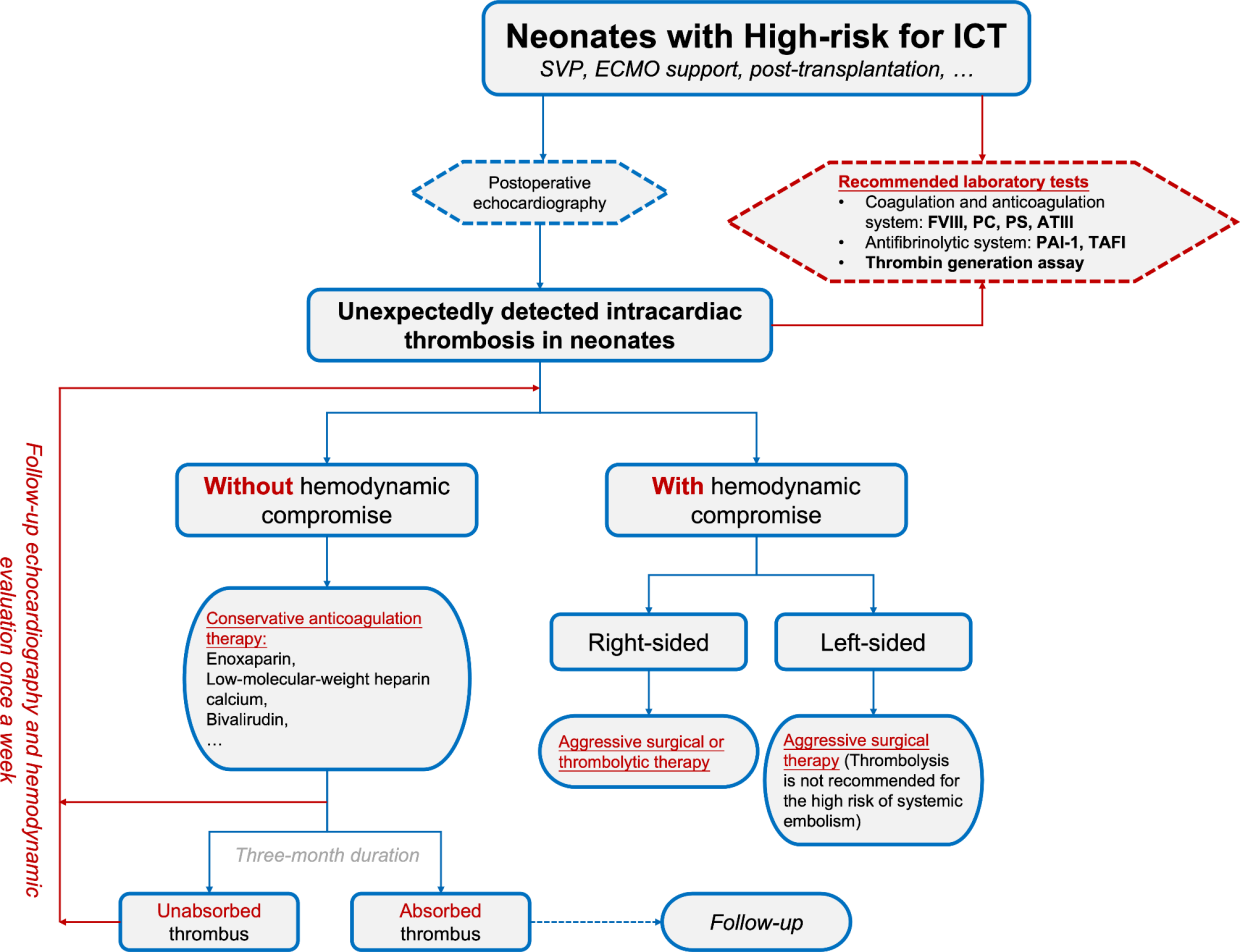
Algorithm of decision-making process for the neonate with intracardiac thrombosis (ICT). SVP, single ventricle physiology; ECMO, extracorporeal membrane oxygenation; FVIII, factor VIII; PC, protein C; PS, protein S; ATIII, antithrombin III; PAI-1, plasminogen activator inhibitor-1; TAFI, thrombin-activatable fibrinolysis inhibitor

Many studies have confirmed single ventricle physiology (SVP), heart transplantation, longer duration of central line use, and postoperative extracorporeal membrane oxygenation (ECMO) as risk factors for postcardiotomy thrombosis in the pediatric population [23, 24], but there is still concern that whether levels of biomarkers in the hemostatic system could predict ICT. S Emarni et al. demonstrated that higher expression of PAI-1 (≥ 15 ng/ml), TAFI (≥ 2.5 µg/mL), and TGA (≥ 300 nM) were predictable to thrombosis in SVP neonatal patients [13]. The similar conclusion was drawn in neonates with other types of defects [14]. Based on these findings, it is necessary to routinely monitor TGA and fibrinolytic factors in high-risk patients for early detection and treatment of ICT (Fig. 3). However, the fluctuation of coagulation and fibrinolytic markers after cardiac operations is always multifactorial and affected by the hypothermic environment during CPB and postoperative inflammation, especially in neonates. The complex geometry and specific hemodynamics could also be inclined to the formation of ICT after complicated intracardiac repairs. Abnormal laboratory results should be explained with caution, combined with the clinical manifestation and echocardiographic evaluation.

There is no generally accepted consensus or guideline for the management of the postoperative ICT in neonates or younger infants. High risk factors for postoperative ICT are defined as SVP, post-transplantation, longer duration of central line use, post-ECMO, implantation of prosthetic devices, and severely reduced LV ejection fraction after the surgery [5, 23–25]. Possible treatments include anticoagulation, surgical thrombectomy, and thrombolysis [1, 26]. Conservative and aggressive strategies were respectively adopted in two cases. Clinical condition might contribute to the decision-making process (Fig. 3). For those open-heart surgeries, in neonates and young infants, after which anticoagulation or antiplatelet are commonly believed unnecessary, special caution should still be taken and center-based protocol could be followed. Although the causes are complex and multifactorial, the early recognition and timely treatment are essential. Weekly echocardiographic recording is recommended for the follow-up of thrombosis events according to the previous research [25].

The case-report with literature review design was the main limitation of this study leading to the weak level of evidence, which was due to the low incidence of ICT in neonates after congenital heart surgeries. The strategy for the management of postoperative ICT was based on our center’s experience and should be verified in the large-scale case-control or prospective cohort study.

Based on our experiences, obstructive physiology, hemodynamic compromise, and deterioration of respiratory state caused by the thrombus are surgical indications. The size of ICT is not a decisive factor in the decision-making process for the surgical treatment, as even relatively large thromboses may not result in obstruction. Surgery and thrombolytic therapies should be discreetly used for the LV thrombus due to the high risk of systemic embolism. Conservative strategy could be applied to the stable patients.

## Data Availability

All data generated or analyzed during this study are included in this published article.

## List of Abbreviations

ASD: Atrial septal defect
CPB: Cardiopulmonary bypass
CT: Computed tomography
CVC: Central venous catheter
IAA: Interrupted aortic arc
ICT: Intracardiac thrombosis
LV: Left ventricle
PCC: Prothrombin complex concentrate
PDA: Patent ductus arteriosus
TAPVC: Total anomalous pulmonary venous connection
TTE: Transthoracic echocardiography
VSD: Ventricular septal defect
